# Height correlates with dyslipidemia in non-overweight middle-aged Japanese men

**DOI:** 10.1186/s40101-016-0119-1

**Published:** 2016-12-06

**Authors:** Yuji Shimizu, Hiroyuki Yoshimine, Mako Nagayoshi, Koichiro Kadota, Kensuke Takahashi, Kiyohiro Izumino, Kenichiro Inoue, Takahiro Maeda

**Affiliations:** 1 Osaka Center for Cancer and Cardiovascular Disease Prevention, Osaka, Japan; 2 Department of Community Medicine, Nagasaki University Graduate School of Biomedical Science, Nagasaki, Japan; 3 Department of Respiratory Medicine, Inoue Hospital, Nagasaki, Japan; 4 Shunkaikai, Inoue Hospital, Nagasaki, Japan; 5 Department of Island and Community Medicine, Nagasaki University Graduate School of Biomedical Science, Nagasaki, Japan

## Abstract

**Background:**

Our previous study showed that height is inversely associated with the risk of stroke in middle-aged Japanese men, particularly in those with a low body mass index (BMI). Since height is regarded as a surrogate maker of childhood social and physical condition, while BMI may reflect primarily on the current physical condition, a detailed analysis of those with a lower BMI may elucidate the effects of childhood conditions. On the other hand, dyslipidemia is recognized as a prominent risk factor for cardiovascular disease. However, no studies have reported on the association between height and dyslipidemia accounting for BMI status.

**Methods:**

We conducted a hospital-based general population cross-sectional study of 3016 Japanese men aged 30–59 years. Dyslipidemia is defined by the Japan Atherosclerosis Society (JAS) Guidelines as follows: triglycerides (TG) ≥ 150 mg/dL and/or low-density lipoprotein-cholesterol (LDL) ≥ 140 mg/dL, and/or high-density lipoprotein-cholesterol (HDL) < 40 mg/dL, and/or lipid lowering medication use.

**Results:**

Independent of classical cardiovascular risk factors, height was found to be inversely associated with dyslipidemia in subjects with a BMI <25 kg/m^2^ but not in subjects with a BMI ≥25 kg/m^2^. Adjusted odds ratios (ORs) and 95% confidence intervals (CIs) of dyslipidemia for an increment of one standard deviation (SD) in height (5.7 cm) were 0.90 (0.82–0.99) for subjects with a BMI < 25 kg/m^2^ and 1.02 (0.89–1.17) for subjects with a BMI ≥ 25 kg/m^2^.

**Conclusion:**

Height is inversely associated with dyslipidemia in those with a BMI < 25 kg/m^2^ but not with a BMI ≥ 25 kg/m^2^.

## Introduction

Height is an easily measured variable and is thought to be determined during childhood and adolescence by genetic predisposition, nutrition, physical, and social environments, as well as other factors [1–3]. Previous studies have reported an inverse association between height and risk of stroke [4–6]. However, cardiovascular risk factors can be regarded as being determined not only by childhood and adolescence but also by current physical and social conditions that are completely independent from height as a risk factor. Previously, we reported an inverse association between height and risk of stroke in middle-aged Japanese subjects with a low body mass index (BMI), suggesting that childhood genetic, social, and physical conditions may contribute to the development of stroke in adulthood since BMI is regarded as a surrogate marker of current physical condition and higher BMI is known to be a classical cardiovascular risk factor [7]. Additionally, dyslipidemia is recognized as a prominent risk factor for cardiovascular disease [8]. However, no studies have reported on the association between height and dyslipidemia accounting for BMI status.

Therefore, we hypothesized that height is inversely associated with dyslipidemia in subjects with a BMI < 25 kg/m^2^ but not in subjects with a BMI ≥ 25 kg/m^2^. To investigate this association, we conducted a hospital-based general population cross-sectional study of Japanese men who participated in a general medical check-up between April 2013 and March 2014.

## Materials and methods

### Study populations

This study was approved by the Ethics Committee for Human Use of Nagasaki University (project registration number 15033079). Written consent forms were available in Japanese to ensure comprehensive understanding of the study objectives, and informed consent was provided by the participants.

The survey population comprised 6645 men aged 30–59 years referred for a general health check-up and recruited in-hospital (Inoue Hospital, Nagasaki, Japan) between April 2013 and March 2014.

Those from whom increased white blood cell (WBC) count data (1223) were not available were excluded. Additionally, to avoid the influence of acute inflammatory disease, those with a WBC ≥ 10000/μL (134 men) were also excluded, as were those from whom BMI data (25 men), serum data (84 men), and interview data (2163 men) were not available, leaving 3016 men participating in this cross-sectional study. There were no differences in cardiovascular risk factors (blood pressure, BMI, and serum data) between participants from whom interview data were available and those from whom it was not.

In Japan, the majority of companies that reach a certain size contact hospital that serve annual health check-ups since such companies have a duty to ensure that their employees receive an annual health check-up. Our present study utilized data and participants from such check-ups, namely, working-age men. We thus concluded that our study population was an accurate representation of the working age population in Japan.

### Data collection and laboratory measurements

Participant height and weight in bare feet and light clothing were measured by an automatic height and body composition analyzer (DC-250, TANITA, Corporation, Tokyo, Japan), and BMI was calculated as weight (kg)/(height (m))^2^.

Trained interviewers obtained information on smoking status, drinking status, and medical history. Fasting blood samples were collected in a siliconized tube. HDL-cholesterol was measured by the direct inhibition method, LDL-cholesterol was measured by a direct method, and triglycerides were measured by an enzymatic colorimetric method (free glycerol elimination). Serum creatinine and fasting blood sugar were measured using standard laboratory procedures. Internal precision management was performed through the use of a quality assurance program troll (SYSMEX CORPORATION, Hyogo, Japan), while external quality control surveillance was conducted through participation in various associations, including the Japan Medical Association (JMA) and the Japanese Association of Medical Technologists (JAMT).

### Statistical analyses

Differences in age-adjusted mean values or prevalence of potential confounding factors by height quartiles were calculated and tested by analysis of covariance. A trend test was performed with a regression model for mean values, and a logistic regression model for proportion. Logistic regression models were used to calculate odds ratios (ORs) and 95% confidence intervals (CIs) to determine the association between height and dyslipidemia. Dyslipidemia was defined by the Japan Atherosclerosis Society (JAS) Guidelines as follows: LDL ≥ 140 mg/dL and/or HDL < 40 mg/dL and/or TG ≥ 150 mg/dL, and/or taking lipid lowering medication [9].

In addition, subjects were stratified by BMI status, since in our previous study, height was found to be inversely associated with incidence of stroke in subjects with a low BMI but not in subjects with a high BMI [7].

Adjustments for confounding factors were made in two ways; first, we adjusted only for age; and second, we included other possible confounding factors, that is, BMI (kg/m^2^), smoking status (never smoker, former smoker, current smoker), alcohol consumption (non-drinker, sometimes drinker, daily drinker), anti-hypertension medication (yes, no), glucose lowering medication (yes, no), systolic blood pressure (mmHg), blood sugar (mg/dL), and serum creatinine (mg/dL).

All statistical analyses were performed with the SAS system for Windows (version 9.3; SAS Inc., Cary, NC). All *p* values for statistical tests were two-tailed, with values of <0.05 regarded as being statistically significant.

## Results

Of the 3016 men in the study, 1952 and 1064 were defined as having a BMI < 25 kg/m^2^ and a BMI ≥ 25 kg/m^2^, respectively.

Among subjects with a BMI < 25 kg/m^2^, 849 (43.5%) showed dyslipidemia, 580 (29.7%) showed LDL ≥ 140 mg/dL, 72 (3.7%) showed HDL < 40 mg/dL. and 376 (19.3%) showed TG ≥ 150 mg/dL. For subjects with a BMI ≥ 25 kg/m^2^, the corresponding values are 739 (69.5%), 427 (40.1%), 132 (12.4%), and 440 (41.4%).

Age-adjusted characteristics of the study population according to height are shown in Table 1. Current drinker status and serum creatinine were significantly positively correlated with height.

**Table 1:** Age-adjusted characteristics of study population in relation to height

	Height quartiles				
	Q1 (low)	Q2	Q3	Q4 (high)	*p* for trend
Median height, cm	164.2	168.4	172.1	177.2	
No. of participants	750	753	752	761	
Age, years	49.3 ± 6.9	47.6 ± 6.9	46.7 ± 7.1	45.8 ± 6.5	
Systolic blood pressure, mmHg	126	126	126	127	0.446
Diastolic blood pressure, mmHg	80	80	81	81	0.220
Body mass index, kg/m^2^	24.1	24.1	24.2	24.1	0.993
Current drinker, %	69.5	72.1	75.8	75.5	0.019
Current smoker, %	38.4	37.7	40.1	37.4	0.696
Anti-hypertensive medication, %	16.0	12.0	14.8	14.2	0.130
Glucose lowering medication, %	3.8	3.5	4.4	3.9	0.851
Lipid lowering medication, %	9.1	8.6	7.4	6.4	0.179
Serum HDL-cholesterol (HDL), mg/dL	59	58	58	57	0.246
Serum LDL-cholestreol (LDL), mg/dL	129	128	127	126	0.212
Serum triglycerides (TG), mg/dL	133	132	126	135	0.365
Blood sugar, mg/dL	100	101	102	101	0.346
Serum creatinine, mg/dL	0.85	0.87	0.91	0.90	0.002

Age: mean ± standard deviation. Height quartiles are <166.7 cm for Q1, 166.7–170.3 cm for Q2, 170.4–174.2 cm for Q3, >174.2 cm for Q4

Table 2 shows the ORs and 95% CIs for dyslipidemia according to height and demonstrates a significant inverse association between these two factors in subjects with BMI < 25 kg/m^2^.

**Table 2:** Odds ratios (ORs) and 95% confidence intervals (CIs) for dyslipidemia in relation to height for total subjects, stratified by BMI status

	Height quartiles				*p* for trend	1 SD increment of height (5.7 cm)
	Q1 (low)	Q2	Q3	Q4 (high)		
Total subjects						
No. of paticipants	750	753	752	761		
No. of cases (%)	426 (56.8)	392 (52.1)	395 (52.5)	375 (49.3)		
Age-adjusted ORs	1.00	0.86 (0.70, 1.05)	0.89 (0.73, 1.09)	0.80 (0.65, 0.98)	0.055	0.95 (0.88, 1.02)
Multivariable ORs	1.00	0.84 (0.68, 1.04)	0.86 (0.69, 1.07)	0.78 (0.62, 0.97)	0.040	0.94 (0.87, 1.01)
BMI < 25 kg/m^2^						
No. of paticipants	485	489	483	495		
No. of cases (%)	237 (48.9)	221 (45.2)	199 (41.2)	192 (38.8)		
Age-adjusted ORs	1.00	0.91 (0.71, 1.17)	0.79 (0.61, 1.02)	0.74 (0.57, 0.95)	0.011	0.91 (0.83, 0.99)
Multivariable ORs	1.00	0.86 (0.66, 1.12)	0.75 (0.57, 0.98)	0.72 (0.55, 0.95)	0.011	0.90 (0.82, 0.99)
BMI ≥ 25 kg/m^2^						
No. of paticipants	265	264	269	266		
No. of cases (%)	189 (71.3)	171 (64.8)	196 (72.9)	183 (68.8)		
Age-adjusted ORs	1.00	0.75 (0.52, 1.08)	1.10 (0.75, 1.61)	0.91 (0.62, 1.32)	0.865	1.02 (0.89, 1.16)
Multivariable ORs	1.00	0.77 (0.53, 1.12)	1.11 (0.75, 1.63)	0.91 (0.62, 1.34)	0.860	1.02 (0.89, 1.17)

*Dyslipidemia* is defined as TG ≥ 150 mg/dL and/or LDL-choresterol ≥ 140 mg/dL, and/or HDL-cholesterol < 40 mg/dL, and/or lipid lowering medication use. *Multivariable ORs* adjusted further for age, systolic blood pressure, antihypertensive medication use, body mass index, smoking status, alcohol consumption, blood sugar, glucose lowering medication use, and serum creatinine. *Height quartiles* are <166.7 cm for Q1, 166.7–170.3 cm for Q2, 170.4–174.2 cm for Q3, >174.2 cm for Q4

Since our study population was compromised of subjects with a BMI < 25 kg/m^2^ (*n* = 1952) and a BMI ≥ 25 kg/m^2^ (*n* = 1064), to avoid the influence of sample size bias on the correlation between height and dyslipidemia, we evaluated the interaction between height and two BMI categories (BMI ≥ 25 kg/m^2^ and BMI < 25 kg/m^2^) on dyslipidemia. Significant interaction between height and BMI category was observed, with a *p* value for the effect of this interaction on dyslipidemia of *p* = 0.049.

Since low BMI (undernutrition) is also regarded as a cardiovascular risk factor [10], status of low BMI might act as a confounding factor on the association between height and dyslipidemia. Therefore, we made additional analysis limited to subjects with normal range of BMI (18.5–24.9 kg/m^2^) and we found essentially same association. The fully adjusted OR and 95% CI of dyslipidemia for an increment of one standard deviation in height (5.55 cm) was 0.88 (0.81, 0.98).

## Discussion

The major finding of the present study was that, independent of classical cardiovascular risk factors, height is inversely associated with dyslipidemia, particularly in those with a BMI < 25 kg/m^2^.

A previous cross-sectional study with 1040 men aged 30–59 years reported a significant inverse correlation between height and total cholesterol [11]. That study is in agreement with our present study demonstrating the inverse association of height and dyslipidemia. We also found further evidence that this significant inverse association is limited to subjects with a BMI < 25 kg/m^2^.

**Fig. 1:**
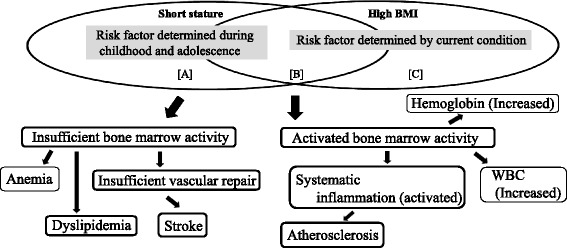
Association between short stature and high BMI as bone marrow activity. **a** Area where potential effects of childhood circumstances can be elucidated. **b** Area where both childhood circumstances and current conditions are included. **c** Area where potential effects of current conditions can be elucidated

The possible mechanism underlying the association between height and dyslipidemia warrants discussion. Height is regarded as a marker of childhood social and physical conditions [4, 7, 12, 13]. On the other hand, BMI is reported to be positively associated with increased risk of disease [14] and is largely influenced by current circumstances. Total cardiovascular risk is likely to comprise a combination of risk factors determined during both childhood and adolescence, as well as risk factors determined by current circumstances. To determine the validity of our hypotheses, we divided the study population into three groups according to height and BMI status (Fig. 1). The first group (a), composed of those with a short stature but normal BMI, was designed to elucidate the potential effect of childhood circumstances as cardiovascular risk. The second group (b), with a short stature and a high BMI, reflecting both childhood circumstances and current conditions, features a higher cardiovascular risk. And the third group (c), with a high BMI but not a short stature, was designed to represent characteristics that could elucidate the potential effect of current conditions. Since there is an association between genetically determined shorter height and increased risk of coronary artery disease, a link that is partly explained by the association between shorter height and an adverse lipids profile [15], childhood circumstances (including genetic factors) (a) may influence the presence of dyslipidemia. Our previous study showing a significant inverse association between height and stroke in subjects with a BMI < 23 kg/m^2^ but not in subjects with a BMI ≥ 23 kg/m^2^ [7] may support these mechanisms.

Since childhood economic hardship was revealed to influence adult height more than adiposity (BMI, waist circumstance, percentage body fat), whereas current economic hardship may be a better determinant of adiposity in Hispanic subjects [16], economic condition might be influenced by the association between height and dyslipidemia. This study also partly supports our above mentioned mechanism since it demonstrates that childhood social condition should influence adult height much more strongly than current BMI status. Furthermore, this study also identified current social status as an important determinant of current BMI status. Further studies that include data on economic status are necessary to clarify the impact of economic status on the association between height and dyslipidemia.

Additionally, bone marrow activity might play an important role in the correlation between height and dyslipidemia. Because bone is an important endocrine organ for the regulation of glucose/lipid metabolism [17, 18], a reduction in bone marrow activity may result in an unfavorable lipid profile such as that seen in dyslipidemia.

Previously, we reported an inverse association between height and high WBC count in subjects with a high BMI (≥23 kg/m^2^) but not subjects with a low BMI (<23 kg/m^2^) [19]. In connection with this mechanism, previous studies have reported a positive association between WBC count and carotid atherosclerosis [20, 21]. We also reported a significant inverse association between height and carotid atherosclerosis in subjects with a BMI ≥ 25 kg/m^2^ but not in subjects with a BMI < 25 kg/m^2^ [13]. In addition, we reported a significant positive association between hemoglobin and increased arterial stiffness [22], while height is inversely associated with normocytic normochromic anemia [23]. Therefore, in subjects with a BMI < 25 kg/m^2^, a positive correlation might exist between height and bone marrow activity since normocytic normochromic anemia may indicate reduced bone marrow activity, whereas an increased hemoglobin level may indicate increased bone marrow activity (hematopoietic activity). Furthermore, we reported in previous study that height is positively associated with reticulocytes, indicating that subjects with a short stature may have lower hematopoietic activity than those with a tall stature [24]. This study also reported that the positive association between height and reticulocytes is particularly relevant for participants with a high hemoglobin level. Height may thus indicate hematopoietic capacity. Those studies may partly support the above mentioned mechanisms.

In our present study, we found a significant positive association between height and current drinker status, as previous studies have also shown [4, 7, 12, 13]. Since alcohol intake influences lipoprotein levels [25], current drinker status may influence the association between height and dyslipidemia. However, when we conducted further analyses evaluating the impact of current drinker status on dyslipidemia, no significant association with dyslipidemia was seen in current drinkers both with a BMI < 25 kg/m^2^ and a BMI ≥ 25 kg/m^2^; the age-adjusted OR of dyslipidemia was 0.83 (0.67, 1.02) *p* = 0.076 for subjects with a BMI < 25 kg/m^2^ and 0.93 (0.7, 1.24) *p* = 0.630 for those with a BMI ≥ 25 kg/m^2^.

Possible limitations of this study warrant consideration. Because creatinine clearance data was not available and estimated glomerular filtration rate (GFR) is not effective for evaluating kidney function when comparing the association with various body heights [7, 13, 26], we were not able to perform an analysis adjusted for precise renal function. However, our study showed that the association between height and dyslipidemia remained significant even after adjustment for serum creatinine. Additionally, since this was a cross-sectional study, we were not able to establish any causal relationships.

## Conclusion

In conclusion, we found that height is inversely associated with dyslipidemia in middle-aged Japanese men with a BMI < 25 kg/m^2^ but not with a BMI ≥ 25 kg/m^2^.

## Abbreviations

BMI: Body mass index
CIs: Confidence intervals
GFR: Estimated glomerular filtration rate
HDL: High-density lipoprotein-cholesterol
LDL: Low-density lipoprotein-cholesterol
ORs: Odds ratios
TG: Triglycerides
WBC: White blood cell
